# Healthy offshore workforce? A qualitative study on offshore wind employees’ occupational strain, health, and coping

**DOI:** 10.1186/s12889-018-5079-4

**Published:** 2018-01-23

**Authors:** Janika Mette, Marcial Velasco Garrido, Volker Harth, Alexandra M. Preisser, Stefanie Mache

**Affiliations:** Institute for Occupational and Maritime Medicine, University Medical Center Hamburg-Eppendorf, Hamburg, Germany

**Keywords:** Offshore wind industry, Occupational strain, Health, Coping strategies, Qualitative analysis

## Abstract

**Background:**

Offshore work has been described as demanding and stressful. Despite this, evidence regarding the occupational strain, health, and coping behaviors of workers in the growing offshore wind industry in Germany is still limited. The purpose of our study was to explore offshore wind employees’ perceptions of occupational strain and health, and to investigate their strategies for dealing with the demands of offshore work.

**Methods:**

We conducted 21 semi-structured telephone interviews with employees in the German offshore wind industry. The interviews were transcribed and analyzed in a deductive-inductive approach following Mayring’s qualitative content analysis.

**Results:**

Workers generally reported good mental and physical health. However, they also stated perceptions of stress at work, fatigue, difficulties detaching from work, and sleeping problems, all to varying extents. In addition, physical health impairment in relation to offshore work, e.g. musculoskeletal and gastrointestinal complaints, was documented. Employees described different strategies for coping with their job demands. The strategies comprised of both problem and emotion-focused approaches, and were classified as either work-related, health-related, or related to seeking social support.

**Conclusions:**

Our study is the first to investigate the occupational strain, health, and coping of workers in the expanding German offshore wind industry. The results offer new insights that can be utilized for future research in this field. In terms of practical implications, the findings suggest that measures should be carried out aimed at reducing occupational strain and health impairment among offshore wind workers. In addition, interventions should be initiated that foster offshore wind workers’ health and empower them to further expand on effective coping strategies at their workplace.

**Electronic supplementary material:**

The online version of this article (10.1186/s12889-018-5079-4) contains supplementary material, which is available to authorized users.

## Background

The offshore wind industry in Germany is a relatively young branch that has rapidly expanded in recent years. In 2015, the industry consisted of approximately 20,500 employees [1], and the number is predicted to increase continuously over time [2]. As a result, more and more employees in Germany are engaged in offshore wind work and thereby confronted with the particulars of this unique work environment (Table 1).

**Table 1:** Work in the German offshore wind industry [66, 82, 83]

Workplace	Offshore wind employees in Germany carry out their work on platforms and installations in the North and Baltic Sea. They transfer to their workplaces by boat or helicopter.
Work organization	The most common work schedule for the workers consists of two weeks offshore followed by two weeks free time onshore (14/14-work schedule). When performing a 14-day offshore turn, workers’ daily work time is 12 h. Shift patterns may include day or night shifts, rotating shifts, and on-call work.
Work tasks	Employees’ work tasks vary according to their specific job types. They may contain demanding physical work, organizational and management activities, or tasks related to offshore workers’ health and safety.
Environmental aspects	The offshore work environment is characterized by its remote location on the high sea, exposing the workers to increased accident risks, changing weather conditions, and particular physicochemical factors, e.g. noise, vibrations, and air-conditioning.
Psychosocial aspects	Psychosocial aspects of offshore work concern the living conditions (e.g. confined spaces, limited privacy, limited opportunities for leisure activities and retreat), as well as the recurrent absences of the workers from home.

Work in offshore settings has been described as demanding, stressful, and potentially dangerous [3–7]. However, because of the young nature of the offshore wind industry in Germany, evidence regarding the specific job demands of its workers is still limited. We recently presented a first empirical approach to the analysis of German offshore wind employees’ working conditions, and identified a broad range of job demands, including hard physical work, long shifts, frequent waiting times, and recurrent periods of absence from home [8]. Likewise, studies from related industries (e.g. the offshore oil and gas industry, seafaring) have revealed similar demands and stressors for workers in these branches [6, 9–15].

### Offshore employees’ occupational strain and health

Offshore wind work requires its employees to be mentally and physically fit [16]. Findings regarding the health of German offshore wind workers are currently restricted to studies analyzing acute incidents of offshore injury and illness from medical evacuation records. These studies found respiratory diseases, flu infections, gastrointestinal problems, headache, and unspecific pain syndromes to be common causes for illness [17, 18]. Further complaints consisted of cardiovascular disease, sleep problems, skin irritations, and general physical and psychological disturbances [17–19]. With regard to the related offshore oil and gas industry, recent studies have found the workers to generally be healthy [20–22]. Due to the required high health standards, it has been discussed that offshore oil and gas workers represent an exceptionally healthy work population [3, 20]. However, evidence is mixed, and health complaints among the workers have also been described (e.g. sleeping problems [3, 13, 22], musculoskeletal complaints [9, 23], and overweight [13, 22, 23]). Likewise, although some studies reported good mental health for offshore oil and gas workers [3, 6, 24], other studies produced conflicting results (e.g. higher levels of nervousness [3], mental fatigue [3, 22], and anxiety [3, 25] in offshore oil and gas workers compared to onshore workers).

Empirical evidence generally suggests that job demands may lead to occupational strain and, as an impairing consequence, to mental and physical health problems [26–29]. When considering offshore employees’ working conditions, there are various potential risk factors that could lead to strain and health impairment of the workers. This, however, has to date not been investigated, at least in regard to the German offshore wind industry. In contrast, a number of studies done on the offshore oil and gas sector has found the demands of offshore oil and gas work to be linked to health impairment of the workers. For example, psychosocial risk factors (e.g. high quantitative demands [24, 30], shift work [31–35], role conflicts [24], and low support at work [30]) were associated with mental and physical health impairment in offshore oil and gas workers [13, 24, 30–34, 36, 37]. Similarly, among seafarers, job demands were associated with fatigue [12, 38] and increased levels of distress [39].

From the above, it can be determined that evidence regarding offshore workers’ occupational strain and health is currently limited. This is especially the case for workers in the German offshore wind industry [40]. Therefore, we aimed to address the following research question:

1. *What are German offshore wind employees’ perceptions of occupational strain and health?*

### Theoretical background

The Job Demands-Resources model (JD-R model) by Demerouti and Bakker [41, 42] provides an adequate theoretical background to aid in answering this research question. According to the model, job demands are defined as aspects of the job that require physical or mental effort and are associated with certain physiological and psychological costs [41, 42]. Job resources are defined as aspects of the job that are functional in achieving work goals, reducing job demands and the associated costs, and stimulating personal development [41, 42]. In the JD-R model, the *health impairment process* suggests that high or unfavorable job demands are positively related to the deterioration of health, whereas the *motivational process* assumes that job resources provide motivational potential and lead to high work engagement [41, 42]. In addition, the model proposes an interaction between job demands and resources, where job resources may buffer the impact of job demands on occupational strain [41]. Occupational strain can be defined as the potential negative responses or consequences to stress and demands at work [43]. Studies provide ample empirical evidence for the main effects of job demands and resources, and considerable evidence for the interaction effects [41].

### Offshore employees’ coping strategies

It has previously been discussed that the impact of working conditions on employees’ health may depend on employees’ coping strategies [44]. Thus, it is likely valid to assume that the use of coping strategies could help offshore workers better deal with their job demands, thereby determining the extent to which they experience strain and health impairment.

Empirical evidence suggests reliable associations between individual coping strategies and health outcomes [45]. In the occupational context, an impact of coping behaviors on the link between working conditions and employees’ health has also been demonstrated [41, 43]. However, investigations have primarily focused on the general working population, and little is known about the coping strategies of workers in offshore industries. Indeed, we were only able to identify two studies, both focusing on the coping behaviors of Chinese offshore oil and gas workers [5, 46]. In one, a beneficial role of problem-focused coping was suggested by indicating a negative association between external and social coping styles and workers’ digestive problems [46]. In the second study, the same group of authors found inconsistent interactive effects between different types of coping and occupational stress on offshore oil and gas workers’ mental health [5], and claimed for further research studies.

It can be concluded that evidence regarding offshore employees’ coping strategies is still limited, in particular concerning German offshore wind workers. Therefore, our second research question was:

2) *What coping strategies do German offshore wind employees use in dealing with the demands of offshore work?*

### Theoretical background

In order to investigate offshore wind employees’ coping behaviors, we adhered to the definition of coping provided by Lazarus and Folkman [47, 48]. Contemporary coping research draws to a large extent on their transactional approach [49], defining coping behaviors as cognitive and behavioral efforts made to master, tolerate, or reduce external and internal demands, as well as conflicts among them [47, 48]. Coping is generally considered to act as a buffer: it interrelates with the stressor and thereby affects the relationship between stressor and health outcome [50, 51]. Coping efforts are initiated when a situation has been cognitively evaluated as potentially stressful (*primary cognitive appraisal*) and available coping resources have been assessed (*secondary cognitive appraisal*) [47, 48]. Since coping may take place on a cognitive, physical, or behavioral level, it is regarded as a multidimensional construct [51]. Coping serves two major functions: the management of the stress-inducing problem (*problem-focused coping*) and the regulation of emotions or distresses (*emotion-focused coping*) [47, 48].

### Study aims

The purpose of our study was to explore German offshore wind employees’ perceptions of occupational strain and health, and to investigate their coping strategies for dealing with the demands of offshore work.

## Methods

### Participants

Twenty one semi-structured telephone interviews with German offshore wind employees were conducted by the first author, a female psychologist (M. Sc.) working as a researcher in occupational health psychology at the time of the study. The qualitative research approach seemed most appropriate to gain initial explorative insights into the topics of interest. The study was carried out within the framework of a funded research project. The study’s results were also intended as a basis for designing a quantitative survey later on. The first author had prior experiences with conducting qualitative interviews and performed a pre-test interview to receive feedback from colleagues and supervisors. We used purposive sampling and reached out to obtain participants from various offshore companies and with diverse sociodemographic characteristics. Inclusion criteria for the interviewees were defined as the following: (1) they had to be fluent in spoken German, (2) had to be at least 18 years old, (3) had to work on a regular 14/14-work schedule, and (4) had to have worked offshore for at least 6 months. In order to publicize the interview study, we sent mails, emails and leaflets to human resources departments, occupational physicians and health and safety managers of German offshore wind companies. We also presented the study at health and safety trainings for offshore wind workers in Germany, encouraging word-of-mouth promotion in the offshore population. Participation in the study was voluntary. All participants received written information about the study and signed a written informed consent prior to the interviews. All interviews were conducted via telephone due to logistical and practicability reasons. Offshore employees were either interviewed during their offshore assignments or during their free turns onshore. No non-participants were present during the interviews. The first author did not know the participants prior to study commencement, and introduced herself to them before starting the interviews. All participants that had initially agreed to be interviewed participated in the study. Interviews were conducted until no new relevant knowledge was being obtained from the interviews, e.g. data saturation was reached. The interviews were conducted in German and were tape recorded. They were from 27 to 60 min in length. Field notes were made immediately after each interview. No repeat interviews were carried out.

### Interview guideline

The interviews were conducted using a semi-structured interview guideline. The guideline was developed to address the research questions within the context of the theoretical background. The interview topic list is illustrated in Table 2. The relevant questions of the guideline are provided in the Additional file 1. The guideline was piloted by an experienced offshore worker who provided valuable suggestions for minor revisions.

**Table 2:** Interview topic list

Introduction	Sociodemographics	Occupational strain and health	Coping strategies
Study information Confidentiality Informed consent	Gender Age Relationship status Occupation Offshore experience Work shifts Living accommodation Project phase	Perceived strain Health impairments Ability to detach from work Fatigue Sleep quality	Strategies for dealing with the job demands

### Analysis

All audio recordings from the interviews were transcribed, anonymized, and double-checked for accuracy. The transcripts were analyzed by the first author using the software MAXQDA Analytics Pro (version 12, VERBI GmbH, 2016). A deductive-inductive approach according to Mayring’s qualitative content analysis was applied. Different codes, categories, and subcategories were derived from the data, iteratively refined, and summarized in a separate document. The findings were profoundly discussed in the team of researchers involved in the study. Discrepancies were talked through until consensus was reached. A process of reflexivity regarding the researchers’ prior assumptions and personal involvement in the study was enhanced during the process of data interpretation. Transcripts and results were not returned to the interviewees. As suggested by van Nes et al. [52], interviewees’ quotes were translated to English with the support of an English native speaker for publication purposes. Moreover, we used the COREQ-checklist (*Consolidated criteria for reporting qualitative research,* [53]) in order to assure reporting quality of our study.

## Results

### Participants

From our study sample, 19 (90.5%) employees were male and 2 (9.5%) were female (Table 3). 11 (52.4%) employees were aged between 31 and 40 years and only 1 (4.8%) was above 50 years old. Employees’ average work experience in the offshore wind industry was 3.4 years (range: 7 months – 8 years). Six workers were technicians (28.6%), followed by 5 (23.8%) workers in quality and maintenance and 4 (19.0%) workers in management positions offshore.

**Table 3:** Participants’ characteristics (*n* = 21)

Variable	Number	Percent
Gender		
Male	19	90.5
Female	2	9.5
Age		
20 − 30 years	5	23.8
31 − 40 years	11	52.4
41 − 50 years	4	19.0
> 50 years	1	4.8
Relationship status		
In a relationship	18	85.7
Single	3	14.3
Occupation		
Technician	6	28.6
Quality, maintenance	5	23.8
Medic	3	14.3
Health and safety	3	14.3
Management offshore	4	19.0
Offshore experience		
< 1 years	2	9.5
1 − 2 years	9	42.9
3 − 4 years	5	23.8
> 4 years	5	23.8
Project phase of wind park		
In construction	5	23.8
In operation	16	76.2
Work shifts		
Day shifts only	15	71.5
Day shifts and flexible night shifts	4	19.0
Rotating shifts (day and night shifts)	2	9.5
Living accommodation		
Offshore on a platform	6	28.7
Offshore on a hotel ship	7	33.3
Offshore on a construction ship	4	19.0
On an island at a hotel or flat	4	19.0

### Offshore wind employees’ occupational strain and health

In the interviews, we identified six major themes regarding offshore wind employees’ occupational strain and health: (1) stress at work, (2) difficulties detaching from work, (3) fatigue, (4) sleep quality, (5) general health and fitness, and (6) physical health impairments.

#### Theme 1:Stress at work

Perceptions of stress at work were reported by most of the employees, although to different extents. Stress perceptions seemed to be related to employees’ specific work tasks, and were more commonly reported by workers with management duties. Moreover, employees stated that higher levels of stress were associated with specific work situations, e.g. during sudden weather changes, unexpected delays in the workflow, or in situations of working under time pressure. The specific consequences of perceived stress on a behavioral level were also communicated. For example, employees reported being more irritable, less team-oriented, and less able to perform their tasks. Some of the workers cited a perceived association between stress at work and the manifestation of physical symptoms, such as headache, fever, or the like:

*“A person who is suffering from mental stress on the platform will get sick. They are no longer productive, they miss shifts, they get a fever or a cold, they get a sore throat or headaches.” [employee #8, age 31-40 years, offshore experience 1-2 years]*

#### Theme 2: Difficulties detaching from work

Interviewees expressed difficulties mentally detaching from work to varying degrees; some reported it to be relatively easy, while others described problems mentally unwinding after a shift during their free time offshore. Job characteristics, such as work tasks, workload, and degree of responsibility, were described to partly determine the chances to detach from work. For example, workers who reported having on-call work and high workloads appeared to struggle more with detaching from work than technicians working on day shifts:

*“Technicians are definitely able to do it [detach from work]. But for electricians or people working in management, they would sometimes stay up all night at their desks, unable to mentally detach from work.” [employee #14, age 31-40 years, offshore experience 3-4 years]*

A major factor cited as contributing to the inability to unwind from work was the lack of spatial separation between employees’ workplaces and living accommodations offshore. This was described as leading to a continued feeling of being in a work atmosphere in the evenings:

*“It’s not really possible to completely ‘switch off’. You are basically [mentally] at work the entire time.” [employee #20, age 31-40 years, offshore experience 1-2 years]*

Additionally, employees indicated organizational factors (e.g. confined spaces, sharing a double cabin) and environmental factors (e.g. noise, vibration) as hindrances to mental disengagement from work. In general, it was stated that unwinding from work was much easier during free time onshore.

#### Theme 3: Fatigue

All of the offshore workers we interviewed mentioned feelings of fatigue during their offshore assignments, albeit to varying extents. The degree of fatigue was described to depend on employees’ work tasks, time, and intensity. Many employees described that the long work time of 12 h caused a state of fatigue and exhaustion. Moreover, climbing up several installations successively on 1 day, working night shifts, or working 14 consecutive days without waiting times in between were reported to contribute to substantial fatigue. Many of the workers noted a particular rise in fatigue following 1 week offshore, increasing steadily thereafter and being especially prevalent during the last days of the turns:

*“You feel good for the first 3 to 4 days. By the last 4 to 5 days, however, you begin to lose steam. You notice you’re much more tired and you can see it in your bloodshot eyes.” [employee #3, age > 50 years, offshore experience 3-4 years]*

During this time, employees reported feeling more tired and less focused and efficient at work, and stated to begin counting the days until departure time. Remarkably, some workers described the sensation of having reached a limit after having spent 14 days offshore:

*“After two weeks, you are ready to go home. It’s like you reach some kind of limit.” [employee #19, age 41-50 years, offshore experience 1-2 years]*

#### Theme 4: Sleep quality

Most of the employees considered the sleep quality offshore to be worse than that onshore, and many workers reported sleeping problems. There were only two workers who stated that they slept equally as well or even better offshore. Different factors contributing to impaired sleep were described, such as permanent noise, sounds, and vibrations on the platforms:

*“It’s definitely a lighter sleep, if not just generally worse. You realize this when you’re home and it feels like you’ve got cotton in your ears; everything is suddenly so quiet.” [employee #21, age 31-40 years, offshore experience 1-2 years]*

Other burdening factors that were mentioned by the interviewees consisted of the confined spaces, unfavorable climatic conditions, and uncomfortable mattresses in the cabins. Employees also described the constant ship movements as being particularly disturbing:

*“And then there’s the constant rocking of the ship; it’s the sea that keeps you awake.” [employee #1, age 20-30 years, offshore experience > 4 years]*

Notably, sleeping problems were especially expressed by employees performing on-call work and/or working on night shifts. Not being able to sleep restfully was regarded as a major cause for increased tiredness and decreased work performance. Few interviewees reported a certain habituation effect and described that they were eventually able to become accustomed to the sleeping conditions offshore.

#### Theme 5: General health and fitness

Overall, interviewees reported being in good general health, and serious cases of illness were reported to be very seldom offshore. Many of the employees pointed out that offshore wind workers were generally supposed to be healthy due to required medical check-ups:

*“There were few problems. You’ve got the offshore examination, anyways. Actually, people were all fit.” [employee #15, age 41-50 years, offshore experience 1-2 years]*

None of the workers reported mental health problems. The fitness level of the workers was also described as being generally good, though varied among the workers. Some of the employees reported that, for some colleagues, overweight was an issue that could complicate physically demanding work. The general health awareness among the workers was described as being mixed; most of the workers reported being distinctly self-aware, but pointed out that others seemed to be less concerned about health issues.

#### Theme 6: Physical health impairments

When asked about physical health impairments experienced in relation to offshore work, the workers cited various adverse associations (Table 4). Most of these were short-term in nature.

**Table 4:** Offshore wind employees’ physical health impairments

Short-term health impairments	
Back problems	
Muscular tension	
Skin problems (dry, rough, chapped hands)	
Knee problems	
Respiratory infections	
Fever	
Headache	
Gastrointestinal complaints	
Seasickness	
Dry mucus membranes	
Long-term health impairments	
Chronic back pain, spinal disc problems	
Chronic muscular pain	
Skin alterations	

Concerns such as muscular tension, back and knee problems were stated by some employees as short-term health complaints due to hard physical work. Other interviewees mentioned the occurrence of respiratory infections (especially in winter months), gastrointestinal symptoms, or complaints such as fever and headache while working offshore. In addition, skin problems (e.g. dry, rough, and chapped hands) and dry mucous membranes due to conditioned air on the platforms were described. Moreover, some workers reported to occasionally experience seasickness while being offshore, e.g. during the transfer to the platforms and installations:

*“If you have to wait a longer time [during transfer] because of marginal weather conditions, it sometimes happens that colleagues experience seasickness.” [employee #10, age 20-30 years, offshore experience 3-4 years]*

Most of the workers stated that they did not expect any long-term adverse health effects as a consequence of offshore work. However, a few employees assumed that specific risk factors of their work environment, such as the exposition to UV radiation, the handling of hazardous materials, or the hard physical work, could contribute to adverse health effects over time. A few interviewees with several years of offshore experience also described adverse health effects they had already experienced, namely chronic back and muscular pain, spinal disc problems, and skin alterations. However, employees themselves questioned whether these concerns could truly be considered as a direct consequence of offshore work.

### Offshore wind employees’ strategies for dealing with the job demands

**Fig. 1:**
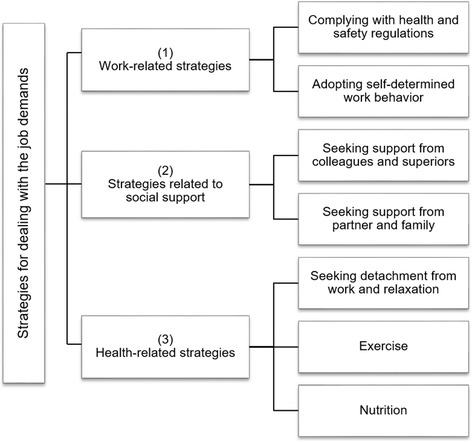
Offshore wind employees’ strategies for dealing with the job demands

When asked about strategies for dealing with the demands of offshore work, employees listed a number of strategies and behaviors which can be classified into three broad categories: (1) work-related strategies, (2) strategies related to social support, and (3) health-related strategies (Fig. 1).

#### Theme 1: Work-related strategies

##### Complying with health and safety regulations

Employees stated that complying with health and safety regulations was crucial in order to deal with potential hazards at the offshore workplace. They emphasized the importance of using personal protective equipment (e.g. survival suits, gloves):

*“When wearing your personal protective equipment, you should not experience any health restrictions.” [employee #18, age 31-40 years, offshore experience > 4 years]*

Moreover, the workers reported using different tools to reduce accident risks (e.g. emery paper on slippery surfaces), carefully following manual handling instructions, and avoiding lifting and carrying extremely heavy loads on their own. In order to cope with the weather conditions, they stated using sun protection on sunny days or wearing warm clothes in the winter months.

##### Adopting self-determined work behavior

Some employees described adopting self-determined work behavior as an important strategy for dealing with their job demands. For example, they reported distributing their work according to their individual scope of action, tailoring their duties to their faculties and capabilities. Some workers explained that they were also able to schedule their own work breaks, which allowed them to better cope with the long 12-h shifts:

*“The freedom to make my own decisions or structure my own work helped. I was always able to organize my tasks in such a way that made it all easier for me to deal with.” [employee #4, age 41-50 years, offshore experience > 4 years]*

Moreover, the workers stated that they were active in the search for new work tasks, becoming involved in decision-making processes. This was described to provide them with a greater sense of control over their work tasks.

#### Theme 2: Strategies related to social support

##### Seeking support from colleagues and superiors

Seeking social support from colleagues and superiors was emphasized by the workers as an important strategy for dealing with their job demands. A strong feeling of comradeship and team support was described:

*“You recognize that you have persons to confide in, persons you can go to when you are feeling stressed and who will talk with you.” [employee #21, age 31-40 years, offshore experience 1-2 years]*

Moreover, time spent with colleagues after work was desribed to alleviate feelings of homesickness. Employees further stated that they felt they could approach their superiors with problems or requests. The presence of a medic on the platforms was also described as a source of support. In line with this, the medics we interviewed described themselves as contact persons for health concerns, but also for private issues and everyday conversations. They defined themselves as *“preventive health providers” [employee #8, age 31-40 years, offshore experience 1-2 years]* and saw their role as taking care of both the physical and mental well-being of the employees.

##### Seeking support from partner and family

Seeking social support from partners and families at home was also described as helpful for coping with offshore work, particularly with regard to the demand of being away from home. According to the interviewees, knowing that their partners agreed with their choice of work facilitated the recurrent absences from home. In general, regular contact with the partners and families at home was regarded as being crucial for the workers’ well-being:

*“That the workers can call home and hear the latest news, or just to make themselves feel better.” [employee #7, age 20-30 years, offshore experience 1-2 years]*

Most employees reported staying in daily contact with their partners while working offshore, keeping them updated about their day-to-day work life. Likewise, they stressed the importance of being informed about what was happening at home when they were away.

#### Theme 3: Health-related stxrategies

##### Seeking detachment from work and relaxation

Many of the interviewees reported strategies to detach from work and relax in an effort to deal with their job demands. For example, some employees described to establish a clear boundary between work and leisure time in order to mentally detach from work, which included avoiding work-related conversations during their free time offshore:

*“We keep [work and free time] as separate as possible. If someone starts to talk about work, then we say, ‘you can deal with that tomorrow, forget about it for now’.” [employee #1, age 20-30, offshore experience > 4 years]*

Other workers highlighted the importance of pursuing calming activities, such as reading, calling home, or watching TV in their cabins. The relevance of having a single cabin in order to relax and withdraw from work was highlighted. Conversely, others reported to pursue energetic activities for relaxation (e.g. exercising), and stressed the relevance of social gatherings after work, such as meeting with colleagues to watch TV together:

*“When you continue to carry out your regular routines on the platform [e.g. watching TV series on Sunday], it’s like bringing a piece of your home life with you. And if you are able to feel like you’re at home, it’s easier to switch off. At least a little.” [employee #8, age 31-40 years, offshore experience 1-2 years]*

##### Exercise

Most of the employees emphasized the importance of exercise in dealing with their job demands. Many of them reported to make regular use of the gym facilities provided on the platforms. Burning off energy was described as a way to balance out the stress of work:

*“If there is the possibility to burn out oneself by doing sports – that helps.” [employee #2, age 31-40 years, offshore experience 3-4 years]*

The possibility to exercise was also cited to be of particular importance (and a welcome distraction) on days when work was impeded due to unfavorable weather conditions.

##### Nutrition

In general, proper nutrition offshore was described as being a crucial factor for employees’ well-being and work performance. Some employees stressed the importance of a needs-oriented diet in staying fit and coping with their job demands. In particular, workers who performed hard physical labor reported the need for a high calorie intake in order to stay fit:

*“You can’t give rope safety technicians just plain bread or a leaf of lettuce. They need ‘real’ food.” [employee #3, age > 50 years, offshore experience 3-4 years]*

Some workers pointed out that the best meals were usually served during the last days of their offshore assignments in order to stabilize the workers’ mood when fatigue began to increase. Moreover, a few employees reported that having occasional coffee breaks at work with sweets provided them with short-term relief in stressful work situations:

*“It’s something you do when you’re feeling stressed; you take a quick coffee break. There are always sweets around, so you can run over and grab a piece of cake or something.” [employee #2, age 31-40 years, offshore experience 3-4 years]*

## Discussion

In our study, we found German offshore wind workers to generally report good mental and physical health. However, occupational strain and health impairments were also stated to varying extents. Moreover, we revealed various coping strategies of the workers for dealing with their job demands. They comprised of both problem and emotion-focused approaches and were either work-related, health-related, or related to seeking social support.

### Offshore wind employees’ occupational strain and health

Referring back to the JD-R model and its proposed health impairment process, our results seem to be in line with the model’s assumption, as the interviewees claimed to experience certain adverse health effects in relation to their job demands. However, when interpreting the results, definite causal conclusions should not be drawn concerning the link between employees’ working conditions and their health; health is generally determined by a complex interplay of multiple factors, so that other variables apart from work (e.g. leisure behaviors, sociodemographic or environmental aspects) likely also play a role in employees’ perceived health. Nevertheless, as we specifically inquired the link between workers’ perceived strain and their *offshore work*, we believe our results to accurately reflect their views on this topic.

#### Stress at work

Most of the interviewees reported perceptions of stress at work, which is consistent with findings for workers in the offshore oil and gas industry [30, 54] and seafaring branch [39]. Our finding of varying stress (with some workers reporting lower levels) agrees with studies in which offshore oil and gas workers also reported rather moderate levels of stress [6, 55]. Moreover, the reported adverse effects of stress (e.g. irritation, decreased work performance) have been similarly shown for offshore oil and gas workers [5, 56].

We found employees’ perceived stress to be related to certain job characteristics (e.g. work tasks, working times). Similarly, differences in stress perceptions have been previously found for different groups of personnel in the offshore sector [39]. A possible explanation might be that different occupational groups in the offshore setting face diverse demands at work, which, in turn, affect the workers’ stress levels differently [20]. Empirical evidence supports this notion; for example, it was shown that sources of stress were specific to certain offshore groups [57], and that perceptions of stressors were dependent on employees’ workplaces (e.g. oil fields, laboratories, offices) [54].

#### Difficulties detaching from work

We found that some offshore wind employees encountered difficulties detaching from work in the evening hours. Based on our literature searches, our study appears to be the first to explicitly address this topic with regard to workers in offshore settings (e.g. wind, oil and gas). Since empirical evidence suggests a lack of detachment to predict high levels of strain [58] and adverse health effects [59], this highlights the importance of this issue for offshore workers.

The lack of spatial separation between the offshore workplace and the offshore residence was described as a main stressor and obstacle for mentally unwinding from work. Consistently, a study done on the general work population has indicated low spatial work-home boundaries to be related to poor psychological detachment from work [60]. Our finding that some workers desired opportunities for seclusion (e.g. single cabins) agrees with a study in which offshore oil and gas workers described a lack of quiet rooms to unwind when off shift as a relevant stressor [57]. Moreover, we found employees reporting high workloads to encounter more difficulties unwinding from work, supporting previous research that showed certain job stressors (e.g. workload) and behaviors (e.g. heavy work investment) to predict of low levels of detachment [58–60].

#### Fatigue

Our finding of fatigue being prevalent among the interviewees correlates well with previous research done in the Norwegian offshore oil and gas sector [22] and the seafaring branch [38]. In accordance with similar findings in these industries [22, 61], workers in our study stated that fatigue increased over time and was especially prevalent during the last days offshore.

The level of fatigue among the workers seemed to be dependent on their job characteristics, with heavy physical work and long working hours being primary reasons for fatigue. Consistently, characteristics of the work environment while on sea (including shift systems, working hours, physical exertion, ship motion and noise) were found to be linked to interrupted sleep and fatigue in seafarers [62, 63]. These risk factors for experiencing fatigue are similarly applicable to the offshore wind workplace. Since fatigue has been associated with short and long-term adverse effects (e.g. reduced performance, sick leave [62, 64]), our results indicate a need for further investigation into this issue.

#### Sleep quality

Our finding that most offshore wind workers experienced impaired sleep quality is consistent with the results from other studies in the offshore wind industry [17], oil and gas industry [3, 13, 31], and seafaring [15, 39]. In line with investigations from the offshore oil and gas sector [32–35], interviewees with on-call and/or night shifts reported to be particularly afflicted with sleeping problems. In addition, interviewees with day shifts also experienced impaired sleep quality, which has been similarly revealed for offshore oil and gas workers who were primarily on day shifts [22]. Furthermore, our finding of experienced tiredness and decreased work performance due to poor sleep supports the notion that abrupt circadian changes can have adverse effects on the sleep, performance, and health of offshore shift workers [13].

Despite there being an already high percentage of sleep problems in the general population [65], our findings reveal certain factors of the offshore environment (e.g. permanent noise, vibrations, and air-conditioning) that may in particular contribute to an increased risk for offshore wind workers of experiencing impaired sleep. Such unfavorable factors seem to be characteristic of offshore settings, as they have also been discussed for offshore oil and gas workers [9] and seafarers [66].

#### General health and fitness

The workers in our study reported good general health and well-being for themselves, agreeing with recent findings for offshore oil and gas workers [20–22]. They also emphasized that offshore wind workers were generally supposed to be in good health due to their medical examinations. Similarly, a ‘healthy worker effect’ has been reported for offshore oil and gas employees [3, 20] and seafarers [63]. For offshore jobs, it seems reasonable that only healthy workers are employed, and that workers suffering from health issues might decide to leave the industry. Thus, a certain positive health bias could be assumed for the German offshore wind workforce.

Our result that only a minority of the offshore wind workers were described as being overweight is somewhat in contrast to findings from the offshore oil and gas industry, where overweight and obesity have been discussed as critical issues [22, 23, 25]. This discrepancy may be explained by the relatively young age of the workers in our study (76.2% were 40 years or younger). In contrast, the average offshore oil and gas worker is in his/her late forties [9]. As the prevalence of being overweight was found to increase with age [67], this may also represent a future health concern for German offshore wind workers, and may even now be prevalent in older workers in this branch.

#### Physical health impairments

Similar to the findings from Bjerkan [20], physical health complaints were more commonly mentioned by the workers in our study than psychological health problems. In contrast to inconsistent results regarding offshore oil and gas workers’ mental health [3, 13, 24], none of the interviewees reported to suffer from anxiety or depressive symptoms. In accordance with results for offshore oil and gas personnel [20, 21, 23, 68], musculoskeletal symptoms made up a substantial majority of the physical health complaints. These symptoms were attributed by the workers to the demanding physical work inherent to their offshore jobs. Other health issues, e.g. respiratory infections and gastrointestinal complaints, have also been previously discussed for workers in the German offshore wind [17] and international oil and gas industry [14]. In addition, we found skin problems and seasickness to be of concern for the interviewees, aspects which have not gained much attention in the literature to date. Our results indicate these complaints to be characteristic of offshore wind work, as interviewees attributed them to their specific work tasks and work environment.

In our study, most of the workers did not foresee any long-term adverse health effects. In a review concerning health risks for Norwegian offshore oil and gas workers, it was concluded that the risk of chronic ill-health as related to offshore work is difficult to estimate [9]; however, musculoskeletal disorders were described as a main reason for chronic long-term ill-health for this workforce [9]. Consistently, a few experienced workers in our study also reported noticeable chronic back or muscular pain as complaints following years of offshore work.

### Offshore wind employees’ strategies for dealing with the job demands

By drawing upon the definition of coping provided by Lazarus and Folkman [47, 48], we identified various strategies used by the employees for dealing with their job demands. As presumed, the use of coping strategies was reported to help the workers to better deal with their job demands. The workers’ strategies comprised both problem-focused and emotion-focused approaches, agreeing with the notion that individuals use both forms of coping in most stressful situations [47].

#### Work-related strategies

Complying with health and safety regulations was reported to be an important strategy in helping the workers deal with potential hazards at work. Due to the particular risks inherent to offshore work, safety concepts have high priority in the offshore wind as well as oil and gas industries [66, 69, 70]. Therefore, it seems reasonable that the workers reported to make a conscious effort to comply with the existing regulations.

Adopting self-determined work behavior (and thereby increasing job control) was also reported as a relevant strategy. This agrees with studies for offshore oil and gas workers, in which high job control was associated with lower levels of mental distress [24], and had a positive influence on workers’ health [20]. Similar positive effects of high job control were also found for seafarers [38] and the general work population [71].

#### Strategies related to seeking social support

Seeking social support from colleagues and superiors represented another relevant coping strategy, which is in line with research showing a positive influence of social coping behavior on offshore oil and gas workers’ health [46]. In addition, further studies in the oil and gas sector have also revealed positive effects of social support on the workers’ health [22, 24, 30, 39, 72]. Seeking social support as a coping strategy has generally received much attention in coping research [73], and reliable positive associations between social support and health outcomes have been revealed in meta-analyses, cross-sectional as well as longitudinal studies [26, 45].

We found that seeking social support from partners and families was another coping strategy for the interviewees, helping them to deal with the absences from home. This corresponds with previous research demonstrating regular contact with people onshore to be vital for offshore wind workers’ well-being [8]. Because the workers spend considerable time away from home, their chances to receive support from partners and families are limited. Therefore, access to means of communication is particularly relevant for the workers, as previously identified [8].

#### Health-related strategies

The health-related strategies mentioned by the interviewees constitute health behaviors that have been found to often be used for coping with stress [74]. When placing workers’ health-related strategies in the context of coping, it should, however, be noted that individuals engage in health behaviors for multiple reasons, only one of which may be coping. As health behaviors are habitual for many people, this makes it difficult to determine whether the reported behaviors can truly be considered as coping strategies [74].

Mentally detaching from work was viewed as an important health-related strategy, agreeing with previous research in which detachment was deemed helpful in the recovery from job demands [59]. Employees’ strategies for unwinding from work included avoiding work-related conversations or activities following a shift. This seems particularly beneficial, since a negative link between the engagement in work-related activities during leisure time and the ability to detach from work has been found [59].

Workers in our study also reported to engage in exercise as a coping strategy, a behavior people frequently use to cope with general and work-related stress [74, 75]. As evidence suggests regular exercise and exercise programs to reduce stress and foster health [74, 76], it seems advantageous for the workers to engage in this strategy.

Moreover, following a needs-oriented nutrition was described as a coping strategy by the workers. This is consistent with findings from general coping research in which eating is listed as a common coping attempt [74]. The importance of appropriate food for offshore workers’ well-being has also been revealed in the offshore oil and gas sector [22, 77]. Notably, workers in our study reported to engage in emotional eating (a general urge to eat in response to negative emotions [74]). Although evidence suggests that emotional eating can lead to longer-term negative health consequences, it also has short-term rewarding and stress-reducing effects [74], which were similarly described by the workers in our study.

In addition, it seems worth noting that only one worker reported sleeping restfully to constitute a coping strategy. This further supports our result of impaired sleep quality offshore, making it difficult for the workers to draw upon this particular coping strategy.

### Strengths and limitations

Our study has several strengths. By focusing on employees in the offshore wind industry, our research addresses a young and innovative occupational field that is becoming of increasing relevance worldwide. Considering the explorative character of our study, another strength consists in our sample size; we conducted 21 interviews, which were sufficient to achieve data saturation [78]. A further strength is the fact that the offshore workers we interviewed differed in sociodemographic variables (e.g. age, occupation, years of offshore experience). Therefore, our results incorporate the views of workers with varying backgrounds who are potentially exposed to varying job demands, which increases the transferability of our findings [79]. All results were thoroughly discussed with the co-authors and compared to empirical references and the theoretical framework, which strengthens the validity of the findings [79]. Furthermore, in order to increase the trustworthiness of our results, we employed rich descriptions of the results and direct quotes from the workers [52].

There remain, however, a few methodological limitations. Due to the qualitative study design and our focus on the German offshore wind branch, our results do not allow for generalizations. Moreover, the applicability of our findings to other offshore settings remains difficult to judge. A further limitation is the fact that we conducted the interviews over telephone, instead of face-to-face. This approach is advantageous in terms of practicability and accessibility, but disadvantageous in its inherent asynchronous communication of place by telephone, resulting in a subsequent reduction of social clues [80, 81]. The workers we interviewed were generally keen to participate in our study, and were predominantly young and healthy. Thus, a potential selection bias cannot be ruled out, and sociodemographic aspects should be taken into account when interpreting the results. In a previous study, offshore oil and gas workers’ health perceptions were found to be influenced by age, with older workers having a more negative perception of their health [20]. As a result, the relatively young average age of the workers in our sample might have contributed to a more favorable rating of their overall health. Furthermore, our study participants had rather little offshore work experience. As offshore oil and gas employees working in the same position for a long time were found to more frequently report health complaints [20], years of offshore experience might also have affected the results.

### Implications

Further research is required in order to generalize our findings and establish a reliable picture regarding offshore wind workers’ strain and health. For future investigations, we recommend that quantitative studies with larger sample sizes be conducted to quantify the actual impact of the working conditions on offshore wind workers’ health. Furthermore, prospective studies should explore the role of factors such as age and offshore experience on offshore wind workers’ health and well-being in the long term.

Practical recommendations can also be derived from our study. Given the fact that occupational strain and health impairment were reported by the offshore wind workers, we conclude that measures should be carried out aimed at reducing their health concerns. Of equal importance, interventions should be initiated that foster and sustain offshore wind workers’ health. For this purpose, and as a first step, employees’ needs for workplace health promotion should be addressed. Subsequently, suitable health promotion interventions should be designed and implemented. Since the workers in our study reported to already engage in coping strategies they considered beneficial for dealing with their job demands, interventions should strive to empower workers to further expand on such effective coping.

## Conclusions

Our study was the first in investigating the occupational strain, health, and coping strategies of workers in the growing German offshore wind industry. By adopting a qualitative research approach, we identified several themes regarding the occupational strain and health of the workers, and revealed their specific coping strategies for dealing with the demands of offshore work. Future research should further investigate the association between offshore wind employees’ working conditions, health, and coping. In addition, health promotion interventions should be initiated that target the reduction of offshore wind employees’ strain, foster their health and empower them to further expand on effective coping strategies at their workplace.

## Additional file

Additional file 1: Questions of the interview guideline. (PDF 182 kb)
