# Supplementary material for: Healthy offshore workforce? A qualitative study on offshore wind employees’ occupational strain, health, and coping
Source: BMC Public Health. 2018 Jan 23;18:172. doi: 10.1186/s12889-018-5079-4 (PMC5781334; doi:10.1186/s12889-018-5079-4)
Supplement: Additional file 1: — Questions of the interview guideline. (PDF 182 kb) [file 12889_2018_5079_MOESM1_ESM.pdf]

## Additional file 1: Questions of the interview guideline

| **Occupational strain and health** |
|---|
| 1. *“Do you feel that your job demands have an impact on your health? If so, what kind of impact?”* |
| 2. *“Are there particular aspects of your work that you would judge as harmful to your health?”* |
| 3. *“Do you view the impacts on your health as rather short-term or long-term?”* |
| 4. *“How do you manage to mentally detach from work after your shifts?”* |
| 5. *“Do you feel that there is a clear boundary between your work times and your free time in the evenings when you’re offshore?”* |
| 6. *“Are there particular phases or days during your offshore assignments when you feel especially fatigued? [psychologically / physically]”* |
| 7. *“How do you rate your sleep quality during your offshore assignments (compared to your normal sleep quality onshore)?”* |
| 8. *“Are there particular aspects of the offshore environment that have a negative or positive influence on your sleep quality?”* |

| **Coping strategies** |
|---|
| 1. *“How do you deal with the demands of your work?”* |
| 2. *“Do you have specific strategies in order to better deal with your job demands?”* |
| 3. *“What do you do to recover from your work when you are offshore?”* |
